# Heading forwards: anterior visceral endoderm migration in patterning the mouse embryo

**DOI:** 10.1098/rstb.2013.0546

**Published:** 2014-12-05

**Authors:** Matthew J. Stower, Shankar Srinivas

**Affiliations:** Department of Physiology, Anatomy and Genetics, University of Oxford, South Parks Road, Oxford OX1 3QX, UK

**Keywords:** anterior visceral endoderm, embryonic patterning, epithelial cell movement, cell migration

## Abstract

The elaboration of anterior–posterior (A–P) pattern is one of the earliest events during development and requires the precisely coordinated action of several players at the level of molecules, cells and tissues. In mammals, it is controlled by a specialized population of migratory extraembryonic epithelial cells, the anterior visceral endoderm (AVE). The AVE is a signalling centre that is responsible for several important patterning events during early development, including specifying the orientation of the A–P axis and the position of the heart with respect to the brain. AVE cells undergo a characteristic stereotypical migration which is crucial to their functions.

## 1. Introduction

In this review, we will cover some of the recent exciting advances to our understanding of the formation and function of the anterior visceral endoderm (AVE). In addition to being important for its role in embryogenesis, the AVE also offers a valuable model to study the control of cell migration in an epithelial context, and in the second half of this review we will focus on our understanding of the cellular machinery that drives AVE cell migration.

## 2. Formation of the anterior visceral endoderm

**Figure 1.**
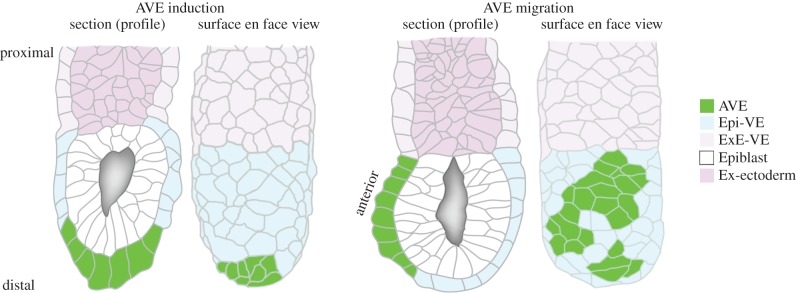
Diagram of E5.5 egg cylinder stage mouse embryos at AVE induction and migration stages showing the major tissues. The AVE migrates unidirectionally from the distal tip to one side of the egg cylinder, thereby defining the anterior (rostral) of the adjacent epiblast. The site of gastrulation (primitive streak) forms on the side of the epiblast opposite to the AVE at E6.5 and generates the three primary germ layers.

Shortly before implantation at around embryonic day (E) 4.5, the mouse blastocyst consists of an outer shell of trophectoderm (TE) enclosing the pluripotent epiblast, visceral endoderm (VE) and parietal endoderm. Implantation stimulates the columnar epithelium of the maternal endometrial wall to completely envelope the conceptus. Coincident with this the conceptus undergoes a profound change in its morphology and size, elongating 2.5-fold along its proximal–distal axis to form the ‘egg cylinder’ [1]. This change in shape is thought to be driven by increased proliferation in the TE and epiblast [2,3], causing them and the overlying VE to grow into the blastocoel cavity. The VE comes to envelope both the proximally located TE-derived extraembryonic ectoderm (ExE) and distally located epiblast (figure 1).

At the distal tip of the egg cylinder a subset of VE cells differentiate into the AVE (also referred to as the distal visceral endoderm (DVE) when at this position). These cells are induced at the distal tip through the interaction of Nodal and MAPK signalling pathways [4–7], become columnar and express characteristic markers including *Lefty1* (left–right determination factor 1), *Cer1* (cerberus-like 1) and *Hex* (haematopoietically expressed homeobox) [8–10]. Although Nodal is expressed throughout the epiblast at this stage, AVE differentiation is restricted to just the distal tip by repressive signals from the ExE [11]. It is believed that the growth of the egg cylinder takes the cells at the distal tip beyond the repressive influence of the ExE, as the AVE is only induced after the egg cylinder is approximately 180 µm long [12].

Hiramatsu *et al.* [13] have recently suggested a role for mechanical stimuli in the induction of the AVE. They reasoned that compressive forces imposed by the uterine tissue surrounding the embryo might have a role in the onset of expression of AVE markers. They tested this by culturing embryos in microfabricated cavities of varying diameter. The majority of E5.0 embryos cultured in narrow cavities (90 µm in diameter) extended along their proximal–distal axis and expressed the AVE marker *Cer1* at the distal tip. By contrast, the majority of embryos cultured in wider cavities (180 µm diameter) elongated to a much lesser extent and did not induce *Cer1*. These experiments suggest that it is the mechanical constraint imposed by the deciduum that is responsible for the elongation of the egg cylinder required for AVE induction.

## 3. Cellular basis for anterior visceral endoderm migration

Seminal DiI labelling experiments by Rosa Beddington and colleagues showed that AVE cells move proximally from their site of formation at the distal tip of the egg cylinder [9] and come to occupy a position diametrically opposite to the site of formation of the primitive streak. Subsequent time-lapse studies of embryos carrying a *Hex-GFP* reporter transgene that marks AVE cells [14] demonstrated that AVE cells migrate actively, sending out cellular projections in the direction of migration [15]. The proximal migratory movement of AVE cells comes to an abrupt halt once they reach the junction between the epiblast and ExE, whereupon they start moving laterally instead, apparently being passively displaced and no longer showing cellular projections [15–18]. The directional migration of AVE cells is central to their function, as failure of migration leads to incorrect patterning and embryonic lethality [4,6,7,12,19–21] (table 1). The endpoint to proximal migration at the junction of the epiblast with the ExE is also presumably important so that AVE cells do not continue to migrate beyond the epiblast and onto the ExE, from where they might be unable to exert a patterning influence on the epiblast.

**Table 1.** Mutations affecting AVE migration and apicobasal polarity. The table lists mutants where the DVE is still induced but arrests at the distal tip or undergoes aberrant or impaired migration; DVE cells are induced but have aberrant apicobasal polarity; and AVE cells overmigrate past the epiblast–extra-embryonic ectoderm boundary. AVE, anterior visceral endoderm; *ActRIB*, activin receptor type IB; BMP, bone morphogenetic protein; *Bmpr1a*, BMP receptor 1a; *Celsr1*, cadherin EGF LAG seven-pass G-type receptor 1 (flamingo homologue 2); *FoxH1*, forkhead box H1; *Ctnnb1*, catenin (cadherin-associated protein) beta 1/(β-catenin); *Ets2*, erythroblastosis virus E26 oncogene homolog 2; *FLRT3*, fibronectin leucine-rich transmembrane protein 3; *Fpn1*, ferroportin 1; *Lefty1*, left–right determination factor 1; *Mpk1*, mouse prickle 1; *Nap1*, Nck-associated protein 1; *Otx2*, orthodenticle homologue 2; *Pten*, phosphatase and tensin homologue on chromosome 10. For a list of mutations that affect induction and patterning of the AVE please refer to Tam *et al.* [22].

gene/allele	modification	pathway or function	AVE phenotype	reference
AVE migration arrested or impaired				
*BMP4*	RNAi knockdown	TGF-β	AVE migration arrest	[23]
*Bmpr1a*	KO	TGF-β	AVE migration arrest	[24]
*Cripto*	KO	TGF-β	AVE migration arrest	[6]
*Foxh1*	KO	Nodal	migration arrest in embryos that induce DVE	[7]
*Ctnnb1* (*β-catenin*)	KO	Wnt signalling	loss of Hex and Hesx cell expression, Cer1 expressed but cells do not migrate	[25]
*Nap1^khlo/khlo^*	KO	activator of WAVE complex	AVE migration severely impaired in half of the mutants	[26]
*Rac1*	KO	Rho-GTPase	AVE migration arrest	[16]
*Pten^M1un^*	KO	phosphoinositide regulation	reduced migration. AVE more dispersed	[27]
*Fpn1 hypomorph*	KO	iron transport	ectopic AVE marker (Cer1) expression at late E5.5 and E6.5. Patterning defects in neural tube. Unclear if migration affected	[28]
*Rab7*	KO	endosome regulation	AVE migration arrest	[29]
Aberrant apicobasal polarity of AVE cells				
*nodal*	KO	Nodal	failure of AVE formation. Highly elongated distal tip cells	[4,10,12]
*ActRIB*	KO	Nodal	loss of apical–basal polarity. Detachment of cells at distal tip	[30]
*Smad4*	KO	TFG-β (Nodal/BMP)	reduced and highly disorganized DVE	[31]
*furin/PACE4* (*Spc1/Spc4*)	KO	Nodal	AVE migration arrest. Highly elongated distal tip	[19]
*Mpk1 (Prickle)*	KO	Wnt–PCP	AVE migration arrest. Epiblast apical–basal poliarty affected	[32]
*Otx2*	KO	transcription factor	AVE migration arrest. Thickening of DVE	[20,33]
*Ets2*	KO	transcription factor	thickening of AVE and partial migration	[34]
*FLRT3*	KO	fibronectin leucine-rich transmembrane protein	highly disorganized basement membrane and rupture of the VE epithelium. Delay in migration and reduced number of Cer-positive cells	[35,36]
Overmigration of AVE				
*Lefty1*	KO	Nodal	overmigration of AVE into ExE-VE	[18]
*ROSA26^Lyn-Celsr1^*	expression of membrane-tethered fragment of Celsr1	Wnt–PCP	overmigration. AVE more dispersed. Whorls of AVE cells	[18]

**Figure 2.**
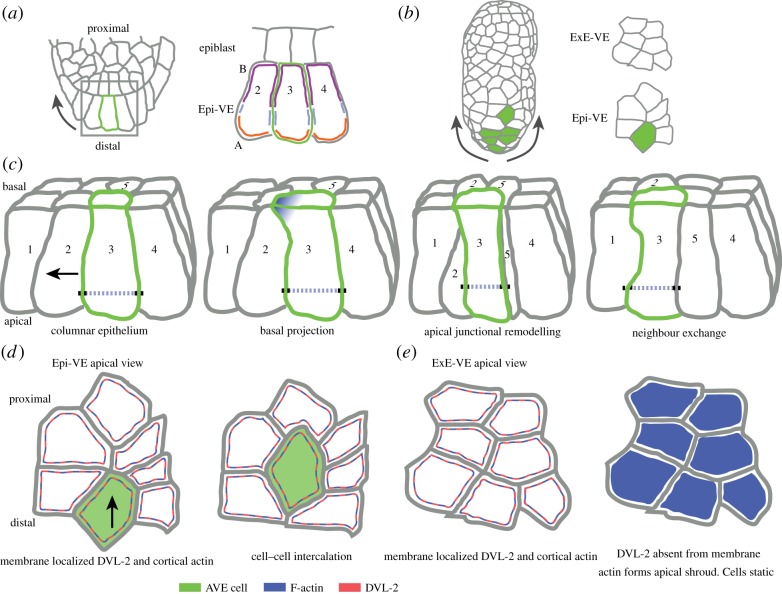
Model of cell–cell intercalation events during AVE migration. (*a*) Diagram of a section of the distal tip of an E5.5 egg cylinder mouse embryo and enlarged region of three columnar Epi-VE cells. One AVE cell is outlined in green which relates to panel (*c*). The apical–basal polarity of the Epi-VE cells is shown via the coloured lines: purple, the basolateral domain; blue, the apical junctional domain; orange, the apicolateral domain. (*b*) En face surface view of mid-migration E5.5 egg cylinder mouse embryo with AVE cells highlighted in green. Clusters of cells have been outlined in the Epi-VE and ExE-VE which relate to (*d*) and (*e*). (*c*) Possible drivers of cell migration events. Diagram of distal tip cells in (*a*) in three-dimensional section profile. Black arrow denotes direction of migration. Black boxes: apical junctional complex. Basal projections driven by Rho-GTPases and the WAVE complex activity are sent out in the direction of migration forming new cell–cell contact sites (blue gradients). Progressively, apical junctional complexes are turned over and remodelled at the leading edge and back of the cell as the cortical actomyosin belt (blue dashed line) drives apical cell shape change to enable cell migration. (*d*) Apical surface view of Epi-VE cells from (*b*) undergoing a directional cell intercalation event. Throughout AVE migration the Wnt–PCP signalling molecule Dishevelled-2 (DVL-2, red line) is strongly localized to the membrane of all Epi-VE cells along with a cortical actomyosin ring (blue line). (*e*) Apical surface view of ExE-VE cells from (*b*) throughout AVE migration stages. Although Dishevelled-2 (red line) and actin–myosin (blue line) are initially localized to a membrane/cortical region prior to migration, Dishevelled is specifically excluded from the membrane and actin forms a ‘shroud’ covering the apical region of ExE-VE cells. Cells are static in this region.

The VE retains epithelial integrity during AVE migration, with intact tight junction (TJ) and adherens junction (AJ) [16,18] (figure 2). Moreover, the VE remains a simple epithelium during the course of AVE migration, meaning AVE cells do not migrate ‘on top’ of other VE cells. Time-lapse studies using differential interference contrast to visualize the apical face of AVE and surrounding VE cells show that AVE cells migrate proximally via directional intercalation, undergoing neighbour exchange (losing contact with a cell or making contact with a new cell) with surrounding VE cells [18] (figure 2*d*). Although the VE is a single continuous epithelial sheet, there are two behaviourally distinct regions. The VE overlying the epiblast (Epi-VE) shows extensive neighbour exchange and cell shape changes, whereas the VE overlying the ExE (ExE-VE) remains largely static and undergoes very few cellular rearrangements [18]. This suggests that AVE cells stop migrating proximally upon reaching the ExE because the ExE-VE is non-permissive to the neighbour exchange events required for migration. Interestingly, mutants with disrupted planar cell polarity (PCP) signalling and *Lefty1* null mutants show an ‘overmigration' phenotype with AVE cells anomalously migrating onto the ExE, indicating that this behavioural difference is regulated by the PCP and TGF-β pathways [18].

**Figure 3.**
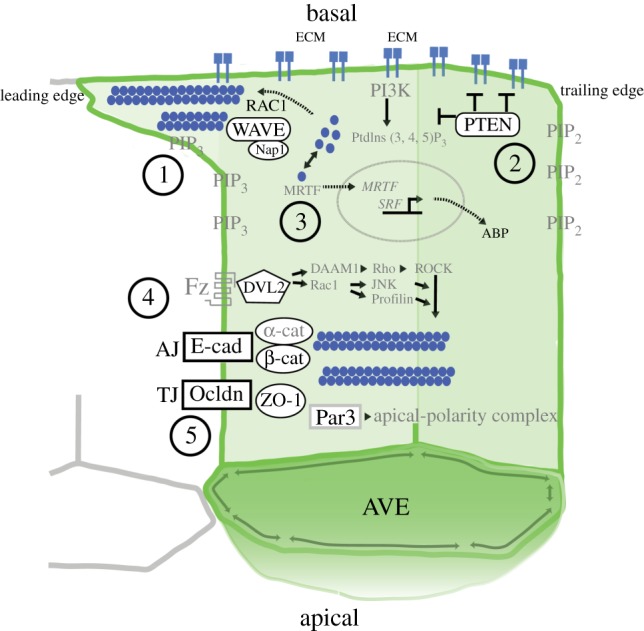
Summary of cellular players active in AVE migration. An AVE cell is depicted with the basal aspect (in contact with the epiblast) towards the top and the apical aspect towards the bottom. Various molecules involved in cell migration are depicted—those with some evidence of a role in AVE migration are shown in black lettering, while those with an inferred role are shown in grey lettering. Different groupings of molecules are labelled with numbers and briefly described below. (1) Cells send out basal projections in the direction of migration (leading edge of the cell) driven by the WAVE complex driving Rho-GTPases [16,26] polymerizing monomeric actin (blue circles) into actin filaments. (2) Cells are polarized along the direction of migration via PTEN [27], which inhibits integrin formation at the trailing edge of the cell and prevents the formation of Ptdlns (3,4,5)P_3_ leading to a phosphatidylinositide gradient in the cell membrane. (3) The status of a cell's actin network is linked to the transcription machinery [40]. Monomeric actin released when F-actin is depolymerized can undergo nuclear translocation with members of the myocardin protein family (MRTFs) [41]. In turn, the MRTF transcriptional cofactors control the activity of serum response factor (SRF) [42], which regulates transcriptional feedback on cytoskeletal and actin-binding protein (ABP) targets. It is interesting to speculate that this system plays a role during AVE migration given that SRF mutant embryos have severe gastrulation defects [43]. (4) Dishevelled is localized to the cell membrane in Epi-VE cells [18] and is an indication of active Wnt–PCP signalling via the Frizzled ligand. Downstream active signalling has been shown to affect actin dynamics via activation of ROCK, RAC and profilin. (5) The AJ and TJ maintain an intact epithelia during migration but are turned over. The AJC provides an interface between cellular polarity and cortical actin. Apical polarity proteins including Par3 and the apical polarity complex (Par6) control the ABP effectors that are permissible in the apical region and control the cell shape change via modulation of the cytoskeleton. BL, basolateral domain; AJC, apical junctional complex; AD, apical domain; AJ, adherens junctions; TJ, tight junctions. Note: only components of the leading edge side shown. Black lettering, published in Epi-VE cells. Grey lettering, hypothesized from other cell systems.

It remains unclear what drives the migratory movement of AVE cells. One possibility is that neighbour exchange in the VE is driven by apical junctional remodelling, as in the intercalation of cells in the *Drosophila* germband [37,38]. This requires the action of non-muscle myosin and sub-cortical actin acting in a coordinated manner across adjacent cells so that certain apical cell edges are contracted and others expanded, ultimately leading to cells exchanging neighbours. However, a different paradigm is offered by the mediolateral intercalation observed during axial elongation in *Xenopus*. Here, cell intercalatory behaviour is driven by medial and basolateral projections sent out by individual cells that draw them together [39]. This system acts in mesenchymal cells of the mesoderm, so at first glance does not seem appropriate to the VE, which is an epithelium. However, there is some support for the possibility of this mechanism acting in the VE. AVE cells show long projections that are up to several cell diameters in length, predominantly in the direction of migration [15]. These projections arise from the basal region of the cell (the portion closest to the epiblast) [16] (figure 2*c*). Moreover, mutants in cellular components like RAC1, PTEN and WAVE, traditionally associated with non-epithelial migration, also show disruption of AVE migration (see below) [16,26,27] (figure 3). This raises the intriguing possibility that some of the molecular mechanisms that mediate migration of individual cells in a mesenchymal context might also be used to regulate migratory behaviour of AVE cells in an epithelial context.

## 4. What controls the direction of anterior visceral endoderm migration?

It has been proposed that the symmetry-breaking event that guides the direction of AVE migration in fact occurs earlier in development, at the preimplantation stage. The expression domains of the AVE markers and Nodal antagonists *Lefty1* and *Cer1* at E5.5 are already tilted towards the prospective anterior *prior* to AVE migration [10]. This is thought to cause an asymmetry in Nodal signalling that provides a directional signal for AVE migration. This is supported by experiments showing that AVE cells will migrate towards ectopically expressed Nodal antagonists [10]. It was initially thought that this asymmetry in *nodal* signalling caused a proliferation difference that nudged the AVE towards the future anterior [10]. However, a more recent study has found no difference in the rate of proliferation in the anterior versus the posterior VE [44], suggesting these Nodal antagonists act by some other mechanism.

Subsequently, both *Lefty1* and *Cer1* were shown to be asymmetrically expressed already in the forming primitive endoderm of the preimplantation blastocyst [17,45,46]. At this stage, the blastocyst is bilaterally (rather than radially) symmetrical because the inner cell mass and the polar TE are tilted with respect to the proximal–distal axis [47,48]. *Cer1* and *Lefty1* expression domains are tilted in the PrE [17,45], and these cells are fated to give rise to the later asymmetrically located *Lefty1-* and *Cerl1*-expressing cells of the E5.5 egg cylinder, pointing to a preimplantation origin for this asymmetrical localization.

Despite evidence that Nodal antagonists control the direction of migrating cells at the distal tip, knockouts of *Lefty1* [49] and *Cer1* [50–52] gastrulate, suggesting the AVE migrates directionally even in the absence of these factors. This could be due to functional redundancy and compensation between these antagonists in single knockouts. Indeed when both *Lefty1* and *Cer1* are removed, more severe anterior–posterior (A–P) patterning phenotypes are shown coincidental with an aberrant accumulation of AVE cells at the prospective anterior [21]. Complicating the picture is the finding that 30% of double mutants have correct streak positioning, suggesting that the AVE migrated successfully in these embryos even in the absence of both Nodal antagonists. The reason for this variability in phenotype is unclear.

Although Nodal antagonists have a major role in controlling AVE migration, there are almost certainly additional mechanisms governing this process. An additional candidate for controlling AVE migration is the secreted Wnt antagonist Dickkopf 1 (DKK1). DKK1 can bind antagonistically to Wnt LRP5 receptors thereby reducing Wnt signalling activity. Kimura-Yoshida *et al.* [53] showed that an exogenous source of DKK1 can act as a guidance cue to AVE cells. Furthermore, *Dkk1* knocked into the *Otx2* locus is sufficient to rescue the AVE arrest phenotype of *Otx2* mutants [53]. Despite such evidence, several questions regarding the role of DKK1 remain to be answered. Firstly, how is a gradient of DKK1 stabilized and maintained as cells in the VE undergo spatial rearrangements? Secondly, although *Dkk1* mutants show loss of expression of the AVE marker *Hesx* at E5.75, *Otx2* is still regionalized to the anterior VE and the primitive streak is correctly positioned [54] suggesting that the AVE is still able to migrate in the absence of DKK1.

In addition to the canonical Wnt pathway, Wnt–PCP pathway also has a role in AVE migration. PCP signalling is a conserved pathway involved in the coordination of multiple morphogenetic processes. For example, the pre-gastrulation chick epiblast undergoes large bilaterally symmetric cellular ‘polonaise’ movements driven by mediolateral cell intercalation events which require the localized expression of the PCP signalling mediators *Flamingo*, *Prickle1* and *Vangl2* [55]. Interestingly, several Wnt–PCP effectors are expressed in the VE including the *Flamingo* homologue *Celsr1* and the *Prickle* homologue *Testin* [56]. *Dishevelled-2* (*Dvl-2*), another component of the core PCP pathway which acts via translocation to the plasma membrane, has also been implicated in AVE migration [18] as the VE shows striking regional differences in its localization. In Epi-VE cells, DVL-2 is localized to the cell membrane, strongly suggestive of active PCP signalling. By contrast, DVL-2 is excluded from the lateral membrane of ExE-VE cells suggesting that active PCP signalling is localized to the regions of the VE where migration occurs (figure 2*d*). Supporting a role for DVL-2 and PCP in control of migratory behaviour, *Lefty1* mutants show ectopic membrane localization of DVL-2 in the ExE-VE, accompanied by abnormal migration of AVE cells into this region [18].

Although core components of PCP and canonical Wnt signalling pathways are often considered to act exclusively, there is evidence in zebrafish that *DKK1*, though traditionally associated with inhibition of the canonical pathway, is also able to act in the PCP pathway. Caneparo *et al.* [57] using antisense morpholino oligonucleotides to knockdown DKK1 in zebrafish embryos noted that this caused an increase in speed of the internalization of the mesendoderm [57]. This effect was shown to be β-catenin independent and dependent upon the binding of DKK1 to the glypican heparan sulfate proteoglycan KNYPEK (KNY) a protein also involved in control of cell polarity during zebrafish convergent-extension movements [58]. Given that DKK1 can act promiscuously in both canonical and PCP pathways, it could be speculated that its role in the context of AVE migration may not be as a traditional guidance cue but rather through the modulation of PCP signalling.

## 5. The anterior visceral endoderm as a multifaceted and changing population

Recent lineage tracing experiments have revealed much greater detail on the origin and fate of AVE cells. *Lefty1* expressing cells marked at E4.5 using a *Lefty1*(*CreER^T2^*) inducible labelling strategy show that these cells contribute mainly to the distal tip of the E5.5 egg cylinder, and at E6.5 to lateral regions of the egg cylinder flanking the anterior [17]. This shows that the AVE cells induced at the distal tip of the egg cylinder at E5.5 first move proximally up to the border of the epiblast with the ExE and then move laterally so that by E6.5 they describe an arc at the Epi-VE/ExE-VE border centred about the prospective anterior. This is consistent with previous time-lapse studies showing that AVE cells of the E5.5 embryo start to move laterally upon reaching the junction of the epiblast with the ExE [15,16].

Interestingly, these lineage labelling studies also establish that at E6.5, the cells in the VE located on the opposite side of the egg cylinder from the primitive streak are different from the cells that occupied this position 24 h earlier, and arise from VE cells at the distal tip of the egg cylinder during the ‘first wave’ of AVE migration at E5.5. This second population of cells appears to follow the first population to the anterior and may be responsible for displacing them laterally.

These findings raise questions about what precisely the definition of the AVE is. We observe that the AVE can be (and has been) defined at different levels:

*Anatomically.* It can be defined as the region of the VE in E5.5–E7.5 embryos that is located opposite where the primitive streak will form or is present. This definition is based on location, so the specific cells occupying this location (and therefore claiming the identity of AVE) can change with time.
*Functionally.* The AVE can be defined as those cells of the VE able to restrict the expression of primitive streak markers like *nodal* and *Cripto* in the epiblast at E5.5 [6,7]. It can also be defined as the cells at E6.5 required for the formation of rostral neural tube derivatives [59], or those cells at E7.5 and later required for the proper positioning of the heart through embryonic folding [60]. The cells carrying out these functions at different embryonic stages are all located in roughly the same anterior anatomical location (see above). However, it is now evident that the cells occupying this location and carrying out these functions change with embryonic stage.
*Marker expression.* The AVE can be defined as cells expressing *Hex*, *Cer1*, *Lefty1*, etc. One problem with this mode of defining the AVE is that these markers do not have identical expression patterns at all stages, blurring the definition. Moreover, both *Hex* and *Cer1* are expressed in a salt-and-pepper pattern in the anatomical AVE at E5.5 [15], with non-expressing cells interspersed among the expressing cells and migrating in a similar manner [18], leading to a lack of identity between the AVE as defined anatomically and as defined by markers.

We suggest it is most useful to think of the AVE in terms of the anatomical definition, which also subsumes the functional definition. In this view, the AVE would be cells in a particular position in the embryo (opposite the primitive streak or the location of the future primitive streak) between approximately E5.5 and E7.5, having the property at various stages of being able to restrict the location of the streak, induce pattern in the epiblast, or position the heart. Therefore, instead of being seen as a fixed or static structure, the AVE should be considered an anatomically distinct signalling centre transiently occupied in the course of development by different cells with various important functions, somewhat analogous to the way the node and primitive streak are structures whose specific cellular make up and function/capability are constantly changing over developmental time.

## 6. The function of the anterior visceral endoderm

### (a) Patterning the epiblast

The function of the AVE has been investigated by microdissection [59], loss of function knockouts [20,21,61] and genetic ablation [62]. Removal of the AVE at E6.5 leads to a loss of forebrain derivatives of the epiblast and a non-viable embryo [59]. However, the AVE is already functional 24 h before this, being essential for the correct positioning of the primitive streak. It does this by restricting the expression of characteristic markers of the primitive streak like *nodal*, *Brachyury* and *Cripto* to the region of the epiblast diametrically opposite it [6,7,63] through the expression of Nodal (*Lefty1*, *Cer1*) and Wnt (*Dkk1*, *Cer1*) antagonists. In knockout embryos in which AVE cells arrest at the distal tip or fail to be induced, the primitive streak is mislocalized in the proximal epiblast and in some cases multiple streaks form, highlighting the key role the AVE has in positioning of the streak and in ensuring that only one streak forms.

The AVE has been described as acting as a head organizer, inducing the expression of rostral markers in the underlying neuroepithlium, but it is not clear that it directly induces anterior pattern rather than protect the epiblast from caudalizing signals [20,64] (reviewed by Stern and Downs [65]). Thus, AVE expression of Nodal and Wnt antagonists, asymmetrically to one side of the epiblast, patterns the epiblast by preventing anterior epiblast cells from undergoing ingression through the primitive streak, through modulation of Nodal signalling, and potentially by extruding extracellular matrix (ECM) components that act to inhibit epithelial to mesenchymal transformation [35].

Grafting experiments in the chick have shown that the hypoblast, the equivalent of the AVE, can transiently induce early ‘pre-neural’ markers including *Sox3* and *ERNI* in the epiblast through the expression of several factors including fibroblast growth factor (FGF) and retinoic acid [66–69]. This suggests that prior to gastrulation, the hypoblast primes cells in the epiblast for their later induction by the node towards a neural fate. In the mouse, the AVE has also been suggested to play a role in inhibiting premature differentiation and retaining pluripotency in the epiblast through *Bmpr1a*-mediated signalling [70]. Although FGF and bone morphogenetic protein (BMP) pathways are often considered to be antagonistic pathways in neural development, it is possible that timing is key to defining context-dependent roles of these signals in directing neural cell fate decisions. Thus, the AVE in coordination with other tissues modulates several major signalling pathways in the epiblast including TGF-β (NODAL, BMP), Wnt and FGF, thereby regulating distinct steps in neural fate acquisition, that is maintenance of pluripotency, prevention of premature differentiation, induction of early ‘pre-neural’ genes, and subsequent prevention of caudalization, while also controlling the timing and spatial restriction of the anterior neural territory.

### (b) Control of epiblast cell movement

In addition to patterning the epiblast, it has been suggested that the chick hypoblast coordinates cell movements in the epiblast prior to the formation of the primitive streak. Rotation of the hypoblast by 90° prior to gastrulation causes the orientation of the primitive streak to bend in the direction of the rotated hypoblast [71,72]. Labelling studies have shown that this phenomenon is not due to a re-specification of fates in the epiblast, but rather that the rotation of the hypoblast alters the pattern of epiblast cell movements [64]. This can be phenocopied by an ectopic source of FGF8, a gene normally expressed in the hypoblast. FGF signalling causes the induction of localized Wnt–PCP signalling that drives intercalation events in the epiblast [55].

At present, it is unknown whether this function of the AVE is conserved between avian and mouse embryos. However, there is some support for the notion of epiblast cells showing directed movement in the finding that *nodal* null embryonic stem cells contribute preferentially to the anterior region of the epiblast in chimaeric embryos [73], suggesting that cell movements in the mouse epiblast may not be completely random.

### (c) Post-gastrulation patterning

The VE was considered to have a role in embryonic morphogenesis up to gastrulation and to contribute thereafter solely to trophic functions of the extraembryonic lineage. However, genetic labelling studies performed by Kwon *et al.* [74] reveal that during gastrulation nascent definitive endoderm cells undergo a widespread intercalation into the VE, forming a single epithelial sheet. These VE derivatives also persist in the gut tube until at least E9.5 [74]. Although it is unknown if this population of cells persists in the adult, these data show that VE cells have additional roles in embryonic development post-gastrulation and that the fetus is not derived exclusively from the epiblast [75].

The AVE also has a role in embryonic folding—the large scale rearrangements during which ventral body wall closure occurs and the embryo internalizes the forming gut. TGF-β signalling from AVE/VE derivatives is required for this process. Tissue-specific knockdown of BMP2 specifically in the VE leads to a striking disorganized-anterior phenotype in 75% of mutants, in which the forming heart remains rostral to the forming brain as a result of folding defect [60,76]. VE specific *Bmp2* mutants also have ectopic neural folds and a loss of the foregut diverticulum, effects which are independent from the AVE role in A–P patterning [60]. Although much is still unknown about the specific mechanism by which the VE directs these morphogenetic events, it is clear that the function of AVE is not confined to just A–P axis formation, and it is likely that it has distinct functions in several developmental processes.

## 7. Cellular mechanism of anterior visceral endoderm migration

For cell movement to take place within an intact epithelium, cell shape needs to continuously change through the active remodelling of the cytoskeleton to drive cell intercalation and neighbour exchange. During this process, the components of AJ and TJ that maintain tissue-wide cohesiveness must be turned over to enable the creation of new contact sites and the restructuring of the cytoskeletal architecture to enable cell movement. In this part of the review, we summarize what is known about cell adhesion, the cytoskeleton and control of cellular polarity in the VE and provide context from other model organisms and *in vitro* studies.

### (a) Polarity and geometry

The apical aspect of VE cells face ‘out’ and form the surface of the egg cylinder, while the apical aspect of epiblast cells face the internal proamniotic cavity. The basal aspects of the VE epithelium and epiblast abut each other, with the ECM of the double basement membrane between them (figure 1). The VE shows regional differences in epithelial morphology around the time of AVE migration. ExE-VE cells are cuboidal with a robust hexagonal apical surface, whereas Epi-VE cells (with the exception of AVE cells) are squamous with greater variation in shape of their apical surface [77]. Interestingly, as the AVE is induced at the distal tip of the egg cylinder, these cells elongate along their apical–basal axis forming a cluster of columnar cells [15,20,78]. It is unclear what precise molecular events drive this characteristic thickening of the VE, although embryos mutant for *nodal* [4,12] and *furin/PACE4* (*Spc1/Spc4*) [19] have an enlarged and elongated distal tip, whose cells appear to have detached from the basement membrane [12]. Furthermore, embryos mutant for the transcription factor *Ets2* have an abnormally thickened AVE at E6.75 [34]. *Ets2* is not expressed in the AVE but is restricted to proximal regions of the embryo (ExE and ectoplacental cone) so must be acting indirectly on AVE cellular morphology. ETS2 is involved in ECM remodelling though the regulation of matrix metalloproteinase expression [79] might be influencing AVE cell morphology by altering the underlying ECM.

It is unclear what the functional implication of the change to a columnar shape is on the migration of AVE cells. However, given that it normally occurs specifically at the distal tip prior to migration, it is tempting to speculate that it is somehow linked to the initiation of migratory behaviour, possibly through the remodelling of the apical–lateral junctional components to enable/enhance cell-intercalation events with surrounding squamous VE.

As cells migrate through neighbour exchange events in the VE there is a change in the geometry of their packing [77]. Prior to migration the packing of cells throughout the VE is orderly with cells having a characteristic shape and number of neighbours. During migration the packing in the Epi-VE shifts towards increased disequilibrium, including the formation of multi-cellular rosettes, groups of five or more cells meeting at a central point [77]. Similar rosettes are associated with convergent-extension movements in both the *Drosophila* embryonic germband [38] and the *Xenopus* kidney tubule [80]. Although rosette formation in the mouse Epi-VE is not associated with convergent-extension movements, mathematical modelling of rosette formation during migration suggests that their formation may be to enhance the orderliness by which cellular rearrangements occur, enabling coherent AVE migration [77].

What is responsible for the formation of multi-cellular rosettes? Interestingly, rosette formation is greatly reduced in ROSA26*^Lyn-Celsr1^* embryos in which Wnt–PCP signalling is affected by the ubiquitous expression of a membrane-tethered C-terminal fragment of the core PCP molecule *Celsr1* [18]. Rosette formation in the *Drosophila* embryonic germband [38] and in *Xenopus* kidney tubules [80] have also been suggested to be PCP-dependent, suggesting that transient PCP-dependent rosette formation may be a general, conserved mechanism controlling context-dependent cell intercalation events facilitating epithelial remodelling.

### (b) Cell adhesion

Simple epithelial tissues such as the VE are characterized by the presence of the AJ. The extracellular domain of epithelial-cadherin (E-cadherin) forms homophilic contacts between clusters of molecules on the surface of adjacent cells, thereby providing the mechanical adhesive force between neighbouring cells by which tissue integrity is maintained. The intracellular domain of E-cadherin interacts with a wide range of protein complexes including α- and β-catenin, which links cell–cell adhesion to the actin–myosin network and cell polarity machinery [81], thereby providing a reference point for the intracellular organization of regulatory components within cells and coordinating their activity across neighbouring cells (figure 3).

E-cadherin is expressed continuously throughout cell–cell junctions in the VE at all stages of AVE migration, confirming that migration occurs within an intact epithelium [16,18]. Although no discontinuity in the level or localization of E-cadherin has been observed among migrating AVE cells or the surrounding VE [18], it is possible that there are cryptic differences in the post-translational modifications to the extracellular domains of cadherin molecules that may provide different adherence properties to each cell type, as has been reported in the *Drosophila* epithelium during dorsal closure [82].

The ubiquity of E-cadherin in the VE of fixed samples almost certainly obscures the dynamics of these molecules; evidence from cell culture and live imaging in *Drosophila* using biotinylation surface labelling [83] and fluorescence recovery after photobleaching with fusion constructs has shown that even in non-motile cells there is a constant turnover of E-cadherin at the cell surface [84]. Depending on the system, turnover is mediated by clatherin, actin or dynamin-dependent endocytosis, that act to remove E-cadherin from the cell surface and recycle it back to the cell membrane [83,85,86]. Cells, therefore, maintain a dynamic equilibrium of E-cadherin at the AJ through continuous recycling and trafficking of E-cadherin between cytoplasmic and plasma membrane pools.

The formation of new E-cadherin cell–cell contacts requires interaction between AJ protein complexes and regulators of the actin cytoskeleton such as members of the Rho family of small GTPases including RhoA, RAC1 and CDC42 [87]. The initiation of new cell contacts requires Rac-mediated actin-based protrusions that carry E-cadherin to new sites where extracellular homophilic E-cadherin contacts can be made and additional complexes subsequently accumulate to expand and stabilize the interaction. Actin-based protrusions initiating new sites of junction formation have been best studied not only in cell sheet morphogenesis, for example, in the converging edges of epithelial sheets during epidermal dorsal closure in *Drosophila* [88], but also seen in the vertebrate wound response [89,90], and also observed in other epithelial re-modelling contexts, for example, in the tracheal branches of *Drosophila* where E-cadherin is localized to filipodial tips which form cell–cell contacts prior to epithelial fusion [91]. To date, although projections have been reported in live culture imaging of migrating AVE cells [15,16,18], fixation of these transient structures has been more difficult so systematic characterization of the cargo carried by these projections has not been possible.

### (c) Cytoskeletal control

The mechanical basis for cellular motility is provided by the dynamic remodelling of the actin–myosin cytoskeleton that controls the mechanical properties of cells and their shape and generates the forces that drive tissue remodelling. Actin fibre networks arise via branching of actin chains and cross-linking of multiple actin filaments. These networks can undergo rapid turnover as filaments are rapidly disassembled. Each of these processes is regulated by specific sets of actin-binding proteins (ABPs). Actin filaments are associated with non-muscle myosin IIA, a motor protein that converts energy from ATP hydrolysis into mechanical work. Assemblies of myosin motors generate contractile tension that can deform cell shape [92,93] by pulling on the anti-parallel actin filaments in the cell cortex.

In the mouse VE, there are regional differences in the localization of actin and myosin IIA; in the Epi-VE, F-actin is localized to cortical rings both before and during AVE migration. By contrast, ExE-VE cells change the localization of their F-actin, initially confined to cortical rings prior to migration, but during and post-migration enriching it to the apical cortex and thereby forming an ‘apical shroud’ of actin. Myosin IIA localization mirrors that of F-actin [18] (figure 2*d*).

Several mouse mutant lines highlight the importance of the actin cytoskeleton for normal AVE migration (table 1). *Rac1* mutants do not undergo AVE migration, fail to form cellular protrusions and show little cell shape change, suggesting that RAC1 plays a major role in the dynamic regulation of the cytoskeleton to bring about cell migration [16]. Similarly, mutants of NAP1, a component of the WAVE complex that acts downstream of RAC1 to control actin branching, also fail to undergo AVE migration in 50% of progeny [26]. While these knockouts have shown the requirement of Rac1 and the WAVE complex for correct migration, at present neither their localization in fixed samples nor their dynamics in living samples have been observed, both of which would be informative for our understanding of actin–myosin regulation in this system.

While RAC1 and the WAVE complex are associated with the leading edge of cells (figure 3), PTEN (phosphatase and tensin homologue on chromosome 10) is associated with controlling the assembly of the cytoskeletal network at the rear of cells by affecting the distribution of phosphatidylinositides (PtdIns) in the plasma membrane [94]. While PtdIns(3,4,5)P_3_ is enriched at the front of migrating cells, PtdIns(3,4)P_2_ is localized to the rear through the action of PTEN that coverts triphosphate PtdIns to their biphosphate form, helping to create a directional gradient of phosphatidylinositides within the cell. This function of PTEN has been associated with controlling the directionality of migrating *Dictyostelium* [95,96], neutrophil cells [97] and the membrane protrusions of axonal growth cones [98]. A similar role has been suggested for PTEN in controlling AVE migration (figure 3). Mouse *Pten*^−/–^ mutants are embryonic lethal at E7.5–8.5 and show AVE migration defects. AVE cells show impaired migration, moving only around 60% of the normal distance. They therefore do not reach their normal proximal position at the boundary between the Epi-VE and ExE-VE, resulting in ectopic streak formation in 20% of mutants [27]. In these mutants, AVE cells also migrate in random directions losing collective organization in the direction of cell movements and becoming more dispersed in the VE. Although PTEN has roles in a wide array of cellular processes, the effects of PTEN loss in the mouse embryo do not appear to be due to changes in proliferation or cell death. Rather cells in the VE have actin mislocalized to the middle of the apical cortex, suggesting that phosphatidylinositides have a role in regulation of the distribution of actin during migration.

## 8. Concluding remarks

The AVE has proved to be a multifaceted tissue with critical functional roles in several important embryonic processes. In addition to its ‘traditional’ role in anterior patterning, it is now shown to be important in the embryonic folding movements required to position the heart correctly. We still understand relatively little about how precisely it carries out these various functions, which remain areas of active research.

Extensive remodelling of epithelial sheets brought about by movement of the component cells underpins key morphogenetic events throughout embryonic development, such as during the formation of the neural tube, branching morphogenesis of the kidneys and formation of sensory organs from epithelial placodes. In addition to being important in patterning the embryo, AVE migration is emerging as a useful model for studying cell movements in an epithelial context. Embryos at this stage can be cultured relatively easily, facilitating detailed time-lapse studies. AVE cells are positioned superficially on the egg cylinder, making them more accessible for imaging and manipulation. Finally, a variety of mutants exist with defective AVE migration, providing a handle on the molecular mechanism controlling this process. Heading forwards, insights from our understanding of AVE migration can be expected to illuminate broader principles of epithelial cell movement.
